# Supplementary material for: Vaccination against PD-L1 with IO103 a Novel Immune Modulatory Vaccine in Basal Cell Carcinoma: A Phase IIa Study
Source: Cancers (Basel). 2021 Feb 22;13(4):911. doi: 10.3390/cancers13040911 (PMC7926323; doi:10.3390/cancers13040911)
Supplement: Supplementary file 1 [file cancers-13-00911-s001.pdf]

Supplementary Material: Vaccination against PD-L1 with IO103 a Novel Immune Modulatory Vaccine in Basal Cell Carcinoma: A Phase IIa Study

Nicolai Grønne Jørgensen, Jeanette Kaae, Jacob Handlos Grauslund, Özcan Met, Signe Ledou Nielsen, Ayako Wakatsuki Pedersen, Inge Marie Svane, Eva Ehrnrooth, Mads Hald Andersen, Claus Zachariae and Lone Skov

Text S1: Flowcytometry on PBMCs

Cryopreserved PBMCs were thawed in wash buffer (0.5% BSA, 2 mM EDTA in PBS) at 37 °C, and Fc-receptors blocked by incubation with human IgG (20 mcg/mL). PBMCs were stained with CD3-FITC, CD56-PE, CD11c-PE, CD8-PerCP, HLA-DR-PerCP, CD27-BV421, CD25-BV421, CD4-BV510, CD28-PE-Cy7, CD3-PE-Cy7, CD19-PE-Cy7, CD127-PE-Cy7, CD45RA-APC, CD56-BV510 (all from BD Bioscience, city, NJ, USA, CCR7-PE, PD-1-APC, CD14-BV421 (all from Biolegend, San Diego, California, , USA), CD16-FITC (Dako, Glostrup, Denmark), and NiR live-dead reagent for APC-Cy7 channel (Invitrogen-Thermo Fischer, Waltham, Massachusetts, USA). A panel for analyzing regulatory T cells (Tregs) with intracellular FoxP3-PE and surface staining CD45RA-FITC, CD4-PerCP, CD127-PE-Cy7, CCR4-APC, CD25-BV421, and CD15s-BV510. Samples were incubated with relevant antibodies for 20 min in the dark at 4 °C. After the cells were washed, acquisition was performed on a FACS Canto II flow cytometer (BD). For intracellular FoxP3 staining, the fixation-permeabilization procedure was performed after surface marker staining, as described for intracellular cytokine staining. Data were analyzed using FACSDiva Software version 8.0.1 (BD Bioscience). Tregs were gated on PBMCs, singlets, live cells, and subsequently on $CD4^+$ cells. $CD4^+$ and $CD8^+$ T cells were characterized by examining live singlet events in the PBMC (lymphocyte and monocyte) gate in the forward and side scatter plot. Naïve T cells were characterized as $CCR7^+CD45RA^+$, central memory as $CCR7^+CD45RA^-$, effector memory as $CCR7^-CD45RA^-$, and effector memory RA^+ as $CCR7^-CD45RA^+$.

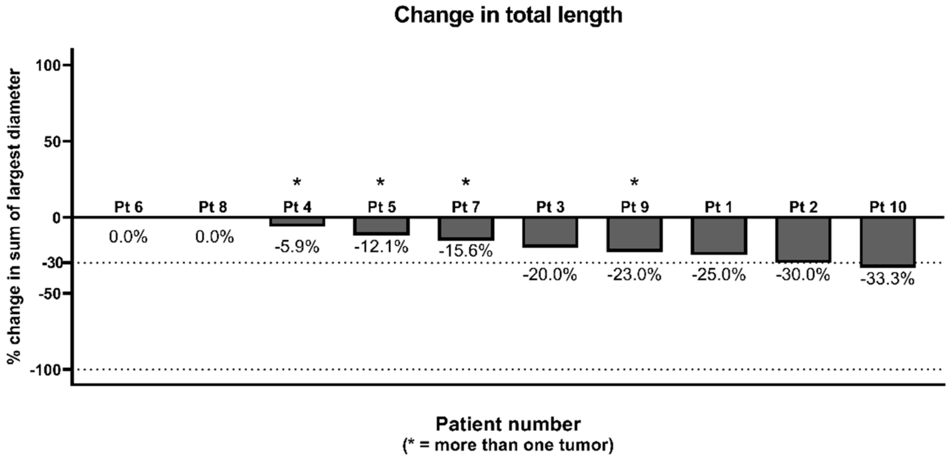

Figure S1. Change in sum of largest diameters of tumors per patient.

A

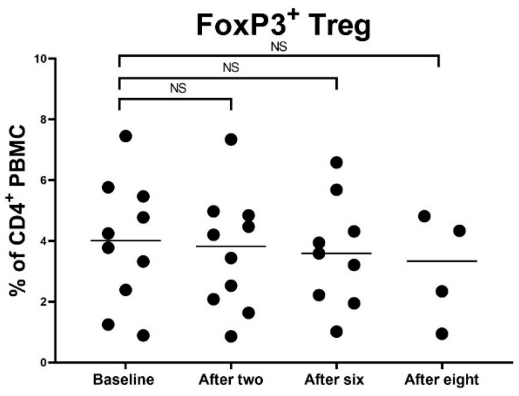

B

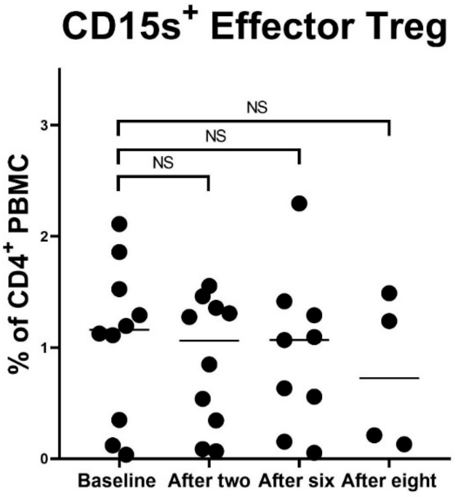

Figure S2. Percentage of regulatory T cells in PBMCs. NS = non-significant.

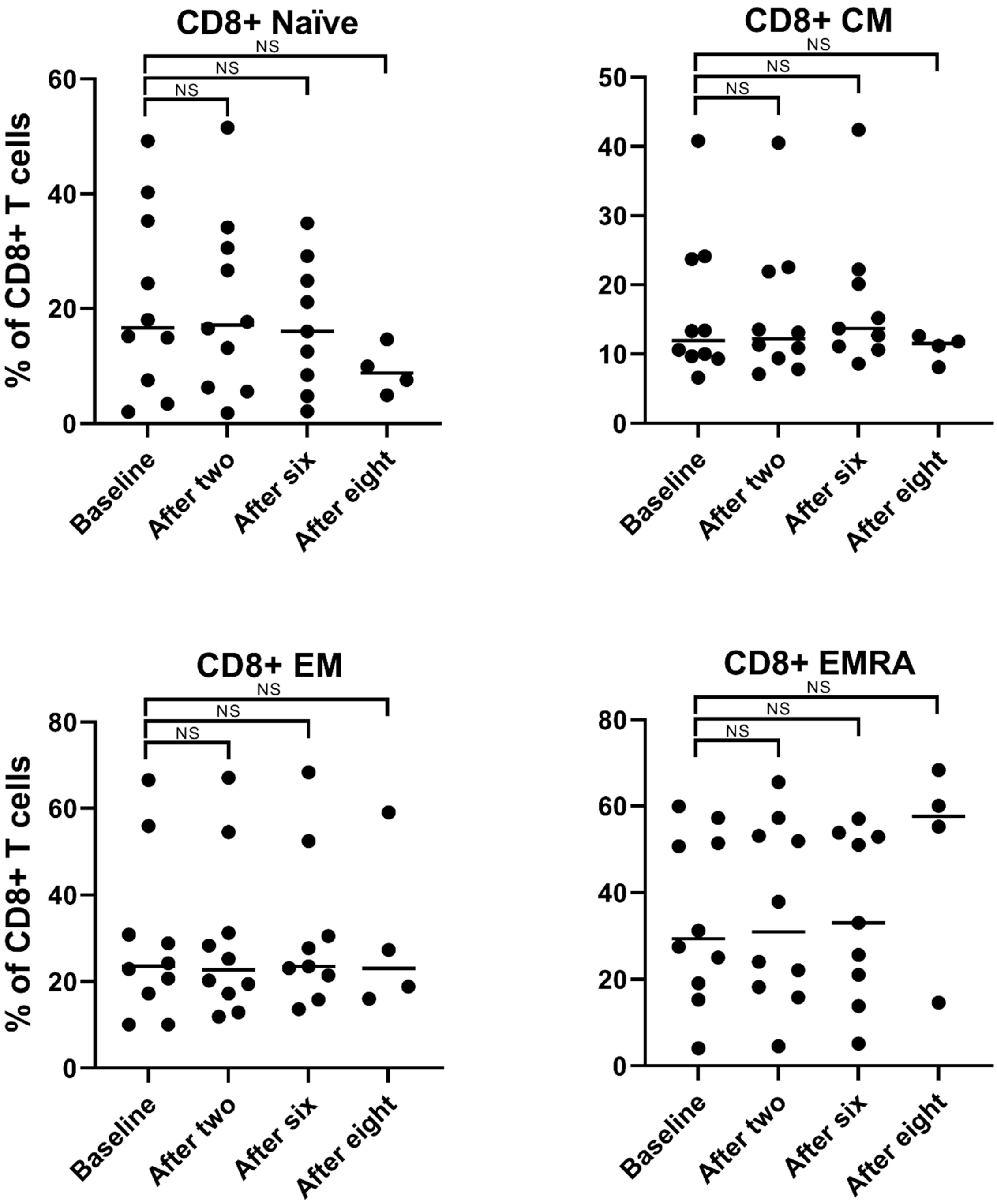

Figure S3. Immunophenotypic CD8+ T cell differentiation. NS = non-significant.

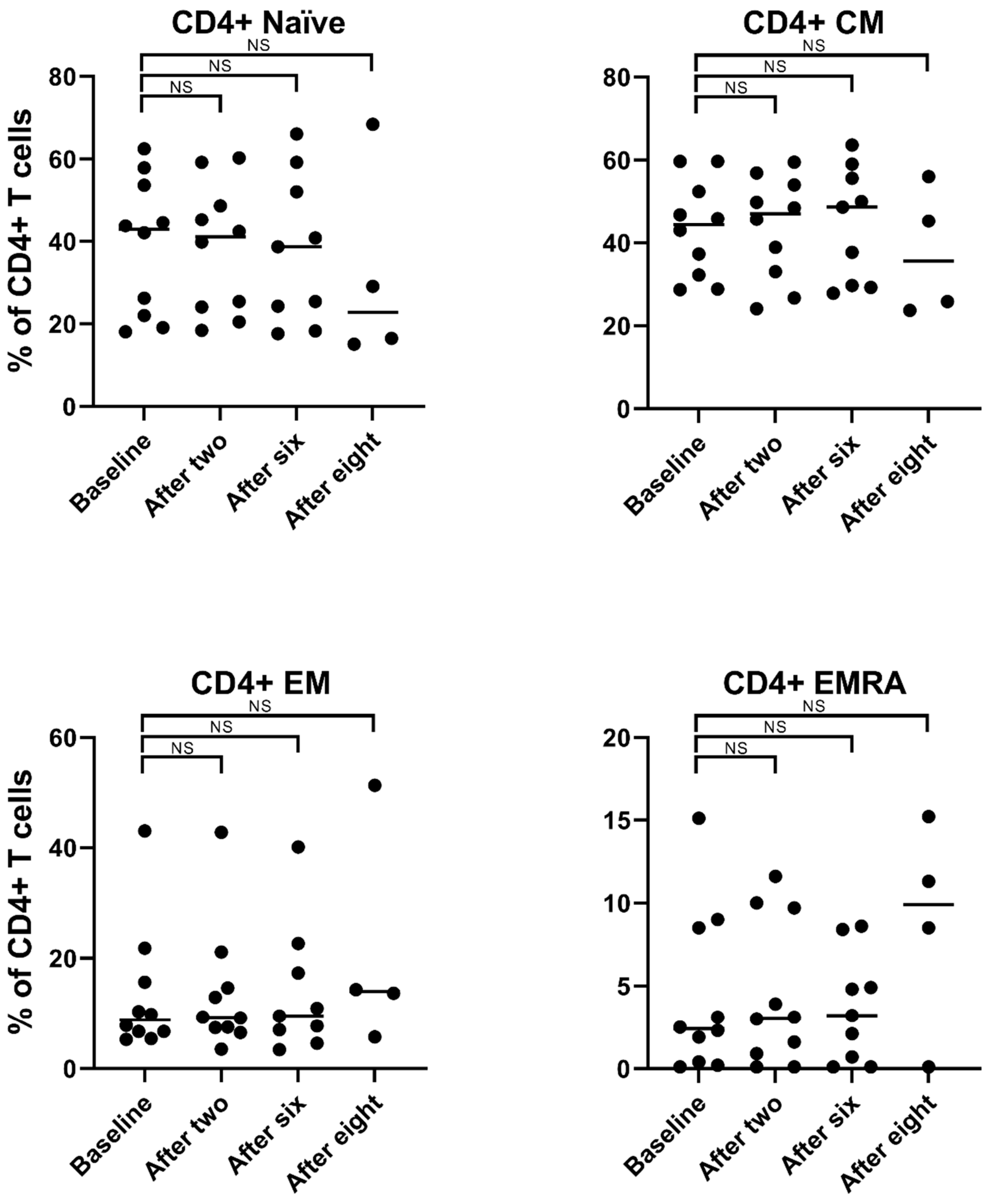

Figure S4. Immunophenotypic CD4 T cell differentiation. NS = non-significant.

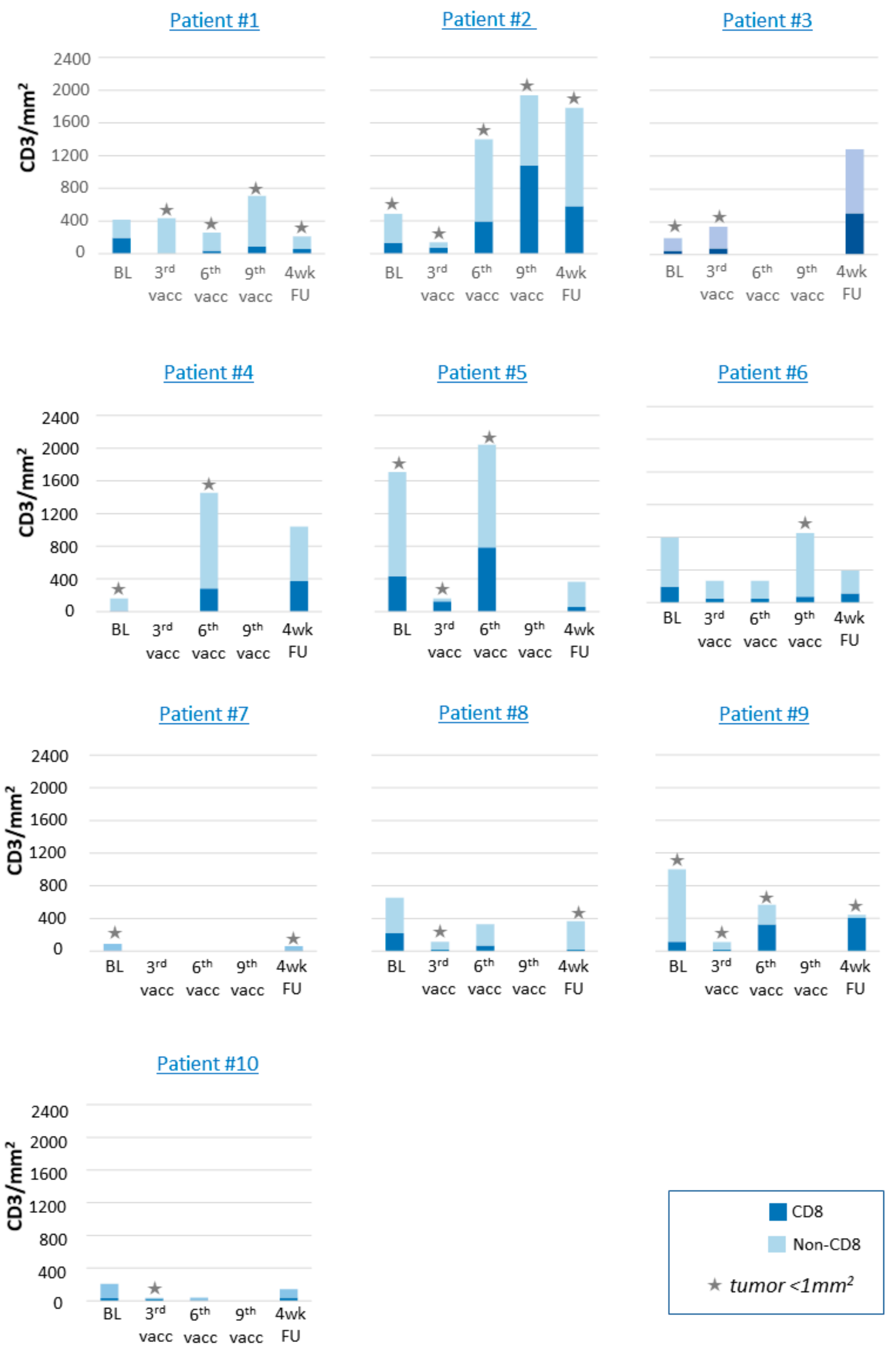

Figure S5. Density of CD3+ and CD8+ cells in sequential biopsies from target tumors. Biopsies were taken at diagnosis, and before 3rd, before 6th, if applicable after 9th and at follow up 4 weeks after last vaccination. Stars: small area (<1 mm²) of tumor cells in the biopsy. BL: baseline.

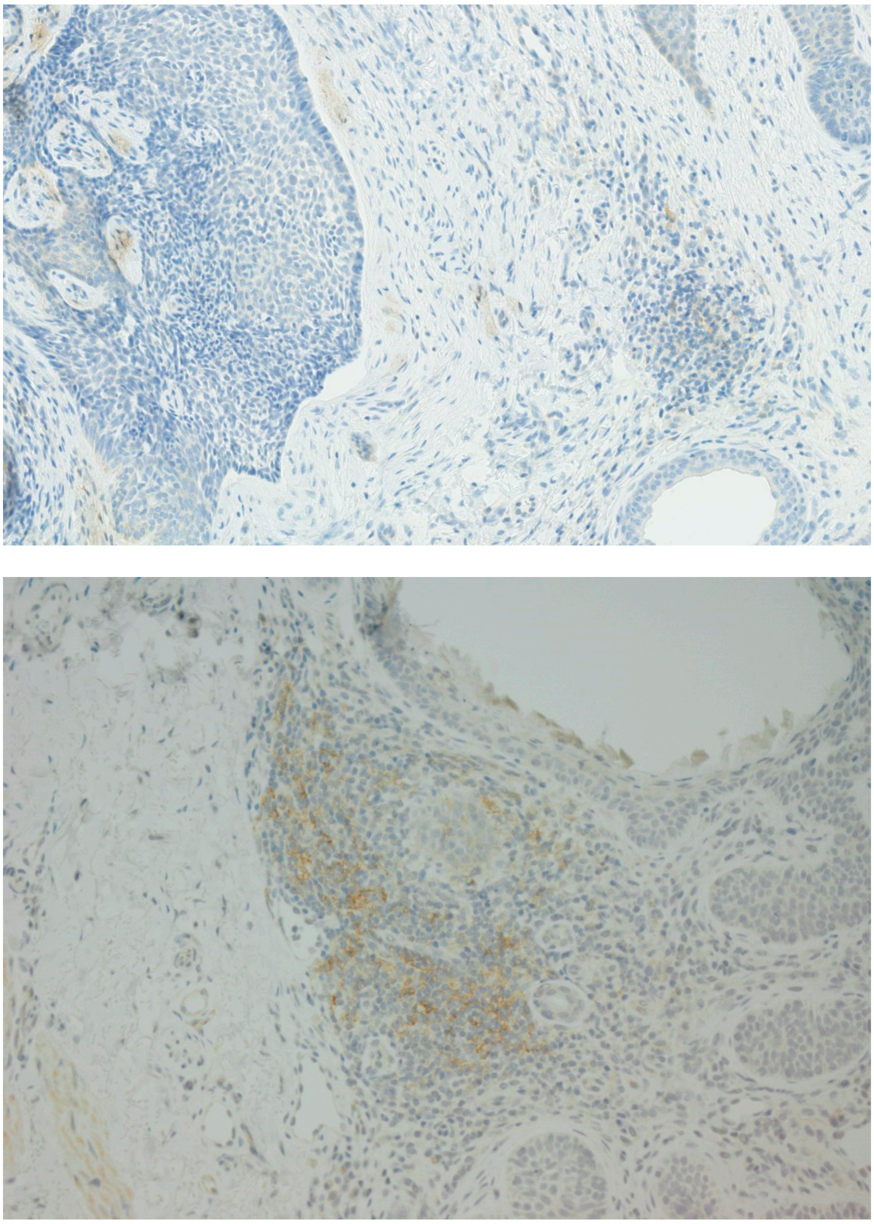

Figure S6. Representative immunohistochemical stainings against PD-L1. Top: Patient #6 at diagnosis. Bottom: Patient #8 at diagnosis. Magnification 20x, anti-PD-L1 clone 28-8.

Table S1: Inclusion and exclusion criteria.

Inclusion criteria	Exclusion criteria
• Age ≥ 18<br>• At least 1 histologically verified superficial or nodular basal cell carcinoma on the body or limbs of bigger than 14 mm in the longest diameter<br>• Willingness to provide three 4 mm biopsies from the lesion/lesions<br>• Not previously treated with a hedgehog pathway inhibitor<br>• For women of childbearing potential: Agreement to use contraceptive methods with a failure rate of <1 % per year during the treatment period and for at least 150 days after the	• A history of life-threatening or severe immune related adverse events on treatment with another immunotherapy<br>• A history of severe clinical autoimmune disease<br>• A history of pneumonitis, organ transplant, human immunodeficiency virus positive, active hepatitis B or hepatitis C<br>• Any condition that will interfere with patient compliance or safety (including but not limited to psychiatric or substance abuse disorders)<br>• Pregnant or breastfeeding patients<br>• Active infection requiring systemic therapy

treatment. Safe contraceptive methods for women are birth control pills, intrauterine device, contraceptive injection, contraceptive implant, contraceptive patch or contraceptive vaginal ring<br>• For men: Agreement to use contraceptive measures and agreement to refrain from donating sperm<br>• The participant provides written informed consent for the trial in accordance with ICH-GCP and local legislation prior to admission to the trial<br>• Sufficient bone marrow function, i.e.,<br>a. Leucocytes ≥ 1.5 × 10^9<br>b. Granulocytes ≥ 1.0 × 10^9<br>c. Thrombocytes ≥ 20 × 10^9<br>• Creatinine < 2.5 upper normal limit, i.e., < 300 µmol/l<br>• Sufficient liver function, i.e.,<br>a. ALAT < 2.5 upper normal limit, i.e., ALAT <112 U/L<br>b. Bilirubin < 30 U/L.	• Vaccination with a live virus vaccine within 30 days of planned start of therapy<br>• Known allergy to Montanide ISA-51<br>• Significant medical disorder according to investigator; e.g., severe asthma or chronic obstructive lung disease, dysregulated heart disease or dysregulated diabetes mellitus<br>• Concurrent treatment with other experimental drugs<br>• Any severe active autoimmune diseases e.g., autoimmune neutropenia, thrombocytopenia or hemolytic anemia, systemic lupus erythematosus, scleroderma, myasthenia gravis, autoimmune glomerulonephritis, autoimmune adrenal deficiency, autoimmune thyroiditis etc.<br>• Severe allergy or anaphylactic reactions earlier in life.

Table S2. All tumor measurements.

	Patient 1	Patient 2	Patient 3	Patient 4	
	Target	Target	Target	Target	Non-target
**At diagnosis**	18x10	13x11	25x15	50 x 30	Not measured
**1st (at enrollment)**	20x18	10x9	25x15	51 x 41	Not measured
**2nd**	20x18	12x10	20x11	50 x 35	Not measured
**3rd**	19x18	10x9	20x11	48 x 35	Not measured
**4th**	16x15	10x9	20x11	48 x 35	Not measured
**5th**	15x15	10x9	20x10	48 x 35	Not measured
**6th**	15x15	10x8	20x10	48 x 35	Not measured
**7th**	15x15	8x7		48 x 35	Not measured
**8th**	15x15	7x5		48 x 33	Not measured
**9th**	15x15	7x5		48 x 30	0 x 0
**Evaluation**	15x15	7x5	20x10	48 x 30	0 x 0

	Patient 5			
	Target	Non-target 1	Non-target 2	Non-target 3
**At diagnosis**	Not measured	Not measured	Not measured	Not measured
**1st (at enrollment)**	19 x 9	15 x 10	13 x 9	11 x 9
**2nd**	18 x 8	15 x 10	13 x 6	11 x 9
**3rd**	18 x 8	15 x 10	13 x 6	11 x 9
**4th**	18 x 8	11 x 10	13 x 6	9 x 9
**5th**	18 x 8	11 x 10	13 x 6	9 x 9
**6th**	18 x 8	11 x 10	13 x 6	9 x 9
**7th**				
**8th**				
**9th**				
**Evaluation**	18 x 8	11 x 10	13 x 6	9 x 9

	Patient 6	Patient 7				Patient 8
	Target	Target	Non-target 1	Non-target 2	Non-target 3	Target
**At diagnosis**	18 x 11	22 x 20	12 x 12	10 x 10	20 x 10	12x10
**1st (at enrollment)**	18 x 11	22 x 20	12 x 12	10 x 10	20 x 10	14x10
**2nd**	18 x 11	20 x 20	12 x 10	9 x 9	20 x 10	14x10
**3rd**	18 x 11	20 x 20	12 x 10	9 x 9	20 x 10	14x10
**4th**	18 x 11	20 x 20	12 x 10	9 x 9	13 x 10	14x9
**5th**	18 x 9	22 x 20	12 x 10	9 x 9	13 x 10	14x9
**6th**	18 x 9	22 x 18	12 x 10	9 x 9	13 x 10	14x9
**7th**	18 x 9					
**8th**	18 x 9					
**9th**	18 x 9					
**Evaluation**	18 x 9	20 x 18	12 x 10	9 x 9	13 x 10	14x9

	Patient 9		Patient 10
	Target	lon-target	Target
**At diagnosis**	20x5	6x6	12x10
**1st (at enrollment)**	20x5	6x6	15x11
**2nd**	20x5	6x6	14x10
**3rd**	20x5	6x6	13x10
**4th**	20x5	6x6	13x10
**5th**	20x5	6x6	13x10
**6th**	20x5	0x0	11x10
**7th**			
**8th**			
**9th**			
**Evaluation**	20x5	0x0	10x9

All measurements are right-angeled longest diameters in millimeter.

Table S3. Total adverse events in the study period**.** Injection site reactions included local erythema, oedema and pruritus. Injection site reactions and flu-like symptoms were deemed related adverse events to the vaccine and were reversible within days. Non-tender subcutaneous lumps up to 1cm in diameter could linger up to months, as is seen commonly with the deposition of the adjuvant Montanide. Deterioration of vision and traumatic injury of eye were in different patients, and not deemed related to the vaccine. AMD: age-related macular degeneration.

Adverse event	Grade 1, n(%)	Grade 2, n (%)
Injection site reaction	10 (100)	
Flu-like symptoms after vaccination	1 (10)	
Deterioration of vision due to worsening known AMD		1 (10)
Traumatic eye injury		1 (10)

Table S4. Target tumor PD-L1 staining and clinical responses in target tumors and non-target tumors.

Patient ID	% PD-L1 (28-8)		BOR (target tumor size reduction)	BOR (non-target tumor size reduction)		
	Tumour	Immune cell		1	2	3
1	0	*	NC (-25%)	-	-	-
2	0	30-40	**PR (-30%)**	-	-	-
3	0	5-10	NC (-20%)	-	-	-
4	0	30-40	NC (-5.9%)	**CR (-100%)**	-	-
5	0	30	NC (+5.3%)	NC (0%)	NC (-18.2%)	NC (-26.7%)
6	0	< 5	NC (0%)	-	-	-
7	0	20*	NC (-9.1%)	NC (0%)	NC (-10%)	**PR (-35%)**
8	0	50	NC (0%)	-	-	-
9	0	< 5	NC (0%)	**CR (-100%)**	-	-
10	0	30-40	**PR (-33%)**	**-**	-	-

% PD-L1: Percentages of cells in biopsies from target tumors positive for immune staining. BOR: best overall response (percent increase or reduction in longest diameter), CR: complete response, NC: no change, PR: partial response.
